# Psychosocial concerns regarding male HPV vaccination

**DOI:** 10.1192/j.eurpsy.2026.10476

**Published:** 2026-07-31

**Authors:** N. Bouayed Abdelmoula, B. Abdelmoula

**Affiliations:** LR23ES07 Genomics of Signalopathies at the service of Precision Medicine, Medical University of Medicine, Sfax, Tunisia

## Abstract

**Introduction:**

The human papillomavirus (HPV) is a widespread sexually transmitted infection affecting both sexes, but vaccination rates for men remain significantly lower than for women. Despite the increasing inclusion of boys in national HPV vaccination programs, numerous psychosocial obstacles continue to hinder acceptance.

**Objectives:**

This study explores the psychosocial concerns influencing the acceptance of the HPV vaccine in boys, young men, their parents and health care providers.

**Methods:**

A comprehensive literature review was conducted using peer-reviewed studies from several countries and cultural contexts. The analysis focused on the knowledge levels, attitudes, beliefs and external influences of parents and healthcare providers. Socio-demographic, cultural and religious factors were also examined.

**Results:**

The low level of awareness and knowledge of HPV in boys and young men contributes to poor uptake of the vaccine, many perceiving a low personal risk. Parents’ misconceptions, such as HPV being a women-only concern or fears of promoting promiscuity-limit vaccination intention, despite generally favorable attitudes. Fathers are generally less informed than mothers, and cost is a constant obstacle in disadvantaged groups. Health professionals support the vaccination of men, but report difficulties in discussing sexual transmission and often prioritize girls in vaccination recommendations. Cultural and religious norms further shape attitudes, especially in communities where sexual health is taboo or abstinence is emphasized. Psychosocial concerns significantly affect HPV vaccination decisions in men. Increased education, culturally appropriate communication and stricter recommendations are essential to overcome these obstacles.

**Conclusions:**

To increase HPV vaccination rates in men, strategies must address the psychosocial dimensions influencing acceptance. Targeted interventions that take into account knowledge gaps, gender norms, cultural sensitivities and health care dynamics are essential for equitable implementation of vaccines.

**Disclosure of Interest:**

None Declared

